# The Mitochondrial GTPase Gem1 Contributes to the Cell Wall Stress Response and Invasive Growth of *Candida albicans*

**DOI:** 10.3389/fmicb.2017.02555

**Published:** 2017-12-20

**Authors:** Barbara Koch, Timothy M. Tucey, Tricia L. Lo, Stevan Novakovic, Peter Boag, Ana Traven

**Affiliations:** ^1^Infection and Immunity Program and the Department of Biochemistry and Molecular Biology, Biomedicine Discovery Institute, Monash University, Clayton, VIC, Australia; ^2^Development and Stem Cells Program and the Department of Biochemistry and Molecular Biology, Biomedicine Discovery Institute, Monash University, Clayton, VIC, Australia

**Keywords:** *Candida albicans*, mitochondria, CEK1, invasive growth, virulence

## Abstract

The interactions of mitochondria with the endoplasmic reticulum (ER) are crucial for maintaining proper mitochondrial morphology, function and dynamics. This enables cells to utilize their mitochondria optimally for energy production and anabolism, and it further provides for metabolic control over developmental decisions. In fungi, a key mechanism by which ER and mitochondria interact is via a membrane tether, the protein complex ERMES (ER-Mitochondria Encounter Structure). In the model yeast *Saccharomyces cerevisiae*, the mitochondrial GTPase Gem1 interacts with ERMES, and it has been proposed to regulate its activity. Here we report on the first characterization of Gem1 in a human fungal pathogen. We show that in *Candida albicans* Gem1 has a dominant role in ensuring proper mitochondrial morphology, and our data is consistent with Gem1 working with ERMES in this role. Mitochondrial respiration and steady state cellular phospholipid homeostasis are not impacted by inactivation of *GEM1* in *C. albicans*. There are two major virulence-related consequences of disrupting mitochondrial morphology by *GEM1* inactivation: *C. albicans* becomes hypersusceptible to cell wall stress, and is unable to grow invasively. In the *gem1*Δ*/*Δ mutant, it is specifically the invasive capacity of hyphae that is compromised, not the ability to transition from yeast to hyphal morphology, and this phenotype is shared with ERMES mutants. As a consequence of the hyphal invasion defect, the *gem1*Δ*/*Δ mutant is drastically hypovirulent in the worm infection model. Activation of the mitogen activated protein (MAP) kinase Cek1 is reduced in the *gem1*Δ*/*Δ mutant, and this function could explain both the susceptibility to cell wall stress and lack of invasive growth. This result establishes a new, respiration-independent mechanism of mitochondrial control over stress signaling and hyphal functions in *C. albicans*. We propose that ER-mitochondria interactions and the ER-Mitochondria Organizing Network (ERMIONE) play important roles in adaptive responses in fungi, in particular cell surface-related mechanisms that drive invasive growth and stress responsive behaviors that support fungal pathogenicity.

## Introduction

Mitochondria are key energy-producing organelles, and they further constitute a platform for integration of metabolic and stress response inputs with cellular growth and developmental decisions. To perform these important functions, mitochondria need to communicate extensively with other cellular compartments, including the nucleus, the endoplasmic reticulum (ER), the vacuole, the plasma membrane and peroxisomes (Murley and Nunnari, 2016; Eisenberg-Bord and Schuldiner, 2017a,b). To achieve this inter-organellar communication, and thereby integrate complex signals to produce growth and development outputs, eukaryotes evolved several mechanisms of organelle-to-organelle interactions. These include metabolites serving as messengers, regulated targeting of proteins to multiple cellular compartments, and membrane to membrane linkages that have been termed “*membrane contact sites*” (Murley and Nunnari, 2016; Eisenberg-Bord and Schuldiner, 2017a,b). Mitochondria utilize all these distinct means of inter-organellar communication, with membrane contact sites playing very prominent functions in physically connecting mitochondria to other organelles.

An important such membrane contact site is facilitated by the ER-Mitochondria Encounter Structure (ERMES), a protein complex that connects the outer membrane of mitochondria with the ER (Kornmann et al., 2009). ERMES-mediated ER-mitochondria contacts are important for several functions. They afford lipid trafficking between the ER and mitochondria, which is essential for the biosynthesis of some lipid species and for providing lipids to mitochondrial membranes (Kornmann et al., 2009; Tan et al., 2013; Ahyoung et al., 2015; Jeong et al., 2016; Kojima et al., 2016). Further to this, ER-mitochondria contacts are important for mitochondrial division, mitochondrial DNA stability and for removing damaged mitochondria through mitophagy (Hobbs et al., 2001; Boldogh et al., 2003; Murley et al., 2013; Böckler and Westermann, 2014). A further key role of ERMES is in enabling tubular mitochondrial morphology (Burgess et al., 1994; Sogo and Yaffe, 1994; Berger et al., 1997; Youngman et al., 2004; Nguyen et al., 2012). Mitochondrial morphology is needed for proper functioning of mitochondrial dynamics—the processes of mitochondrial division and fusion, as well as mitochondrial movement in cells and contacts with other organelles, all of which enable cells to utilize their mitochondria optimally to drive energy production and stress survival, and therefore preserve cellular fitness and viability (Friedman and Nunnari, 2014; Labbé et al., 2014). The importance of ER-mitochondria connections is further underscored by the fact that ERMES has been proposed to be a part of a larger organizing center called ERMIONE (ER-Mitochondria Organizing Network) (van der Laan et al., 2012). The model proposes that ERMIONE is nucleated by ERMES and MINOS (Mitochondrial Inner Membrane Organizing System), a protein complex that connects the inner and outer membranes of mitochondria (Harner et al., 2011; von Der Malsburg et al., 2011). ERMIONE further brings together the TOM (Translocase of the Outer Membrane) and SAM (Sorting and Assembly Machinery) complexes that function in mitochondrial biogenesis (protein import and assembly of outer membrane proteins respectively), as well as several other mitochondrial proteins (van der Laan et al., 2012). In this way, ERMIONE plays a global role in orchestrating ER-mitochondria interactions, thereby controlling mitochondrial morphology, lipid homeostasis, and organelle biogenesis.

We are interested in mitochondrial roles in human fungal pathogens, particularly in relation to pathways required for virulence, and the prospect of acting on mitochondria with new antifungal drugs. With the increasing involvement of fungi in life-threatening human infections, and an estimated 1.5 million deaths annually from invasive fungal diseases (Brown et al., 2012), it is crucial that we expand the very limited repertoire of antifungal treatments. Given their central roles in mitochondrial biogenesis and function, ER-Mitochondria contact complexes are of interest to characterize in fungal pathogens (Calderone et al., 2015). They are also significant because during evolution ERMES has been lost in metazoans, and therefore no human homologs exist (Wideman et al., 2013), and in the human pathogenic yeast *Candida albicans* repression of *MMM1*, the gene encoding the ER-anchored subunit of the ERMES, blocks virulence in mice infection (Becker et al., 2010). We recently performed the first detailed characterization of the ERMES complex in a human fungal pathogen, showing that the principal role of ERMES in *C. albicans* is to enable proper mitochondrial morphology (Tucey et al., 2016). We further demonstrated that inactivation of ERMES crippled the ability of *C. albicans* to evade innate immunity by macrophages, and further led to loss of fitness, particularly at host temperature of 37°C and particularly after long term, chronic inactivation of ERMES function in gene deletion mutants (Tucey et al., 2016). Genetic and cell biology data support the existence of an ERMES complex in *C. albicans* that is of equivalent composition as in *S. cerevisiae*, with four core subunits: Mmm1, Mdm10, Mdm34, and Mdm12 (Kornmann et al., 2009; Tucey et al., 2016). ERMES is conserved within fungi (Wideman et al., 2013), and a recent study of ERMES in the pathogenic mold *Aspergillus fumigatus* supports the idea that targeting ER-Mitochondria interactions might be promising as a therapeutic strategy against multiple, divergent pathogenic fungal species (Geißel et al., 2017).

How the functions of ERMES, and more globally ERMIONE, are regulated to meet the energetic and metabolic demands of cells, is poorly understood. The first evidence showing that ERMES activity might be regulated came from the identification of a fifth subunit of *S. cerevisiae* ERMES, the GTPase Gem1 (Kornmann et al., 2011; Stroud et al., 2011). Gem1 is the fungal ortholog of metazoan Miro, a calcium binding GTPase required for the movement of mitochondria (Tang, 2015; Kanfer and Kornmann, 2016). Early studies of Gem1 in *S. cerevisiae* revealed roles in mitochondrial morphology and inheritance of mitochondria during cell division, but the mechanism remained elusive (Frederick et al., 2004, 2008). Gem1 is located in the mitochondrial outer membrane (Frederick et al., 2004), and recent biochemical identification of Gem1 as interacting with the ERMES complex provided a potential explanation for the mechanism of action of this GTPase in yeast (Kornmann et al., 2011; Stroud et al., 2011). While in *S. cerevisiae* Gem1 does not control ERMES complex formation (Kornmann et al., 2011; Nguyen et al., 2012), it has been proposed to be a regulator of ERMES functions in lipid homeostasis (Kornmann et al., 2011), although the roles of Gem1 in lipid trafficking have been questioned (Nguyen et al., 2012). *S. cerevisiae* Gem1 has further been proposed to regulate ERMES functions in the context of ER-associated mitochondrial division, in which Gem1 promotes organelle separation by loosening ER-mitochondria interactions (Murley et al., 2013).

While the mentioned studies provide a framework for understanding the roles of Gem1 in fungal biology, the full spectrum of cellular functions of Gem1 in fungi, and its mechanism of action, are still poorly characterized. Moreover, how regulators of ER-mitochondria contact sites, such as Gem1, might impact on fungal virulence-related biology has not been studied so far. We therefore undertook a study of the *C. albicans* Gem1. Our results show that, in *C. albicans* the predominant role of Gem1 is in ensuring proper mitochondrial network morphology. We further show that Gem1 is needed to establish virulence determinants that enable invasive *C. albicans* infections. Although a direct physical interaction between Gem1 and the ERMES complex has yet to be determined in *C. albicans*, our phenotypic analysis shows that the *gem1*Δ*/*Δ and ERMES mutants share multiple phenotypes, supportive of a coordinated function of Gem1 and ERMES in this fungal species. We discuss the significance of our study for understanding how mitochondrial dynamics drives fungal pathogenicity mechanisms.

## Materials and methods

### Strains and growth conditions

The *C. albicans* strains used in the study are described in Table 1. The *gem1*Δ*/*Δ mutant was constructed in the BWP17 background using PCR and homologous recombination using the *URA3* and *ARG4* markers to replace the two copies of the *GEM1* open reading frame. Histidine prototrophy was restored by integrating the *HIS1*-containing integrative pDDB78 vector, into the endogenous *HIS1* locus. The same vector was used to construct the complemented strain, by introducing a wild type *GEM1* gene under the control of its own promoter. The conditional *mdm12*↓ was constructed by deleting one copy of the gene utilizing the *ARG4* marker and placing the other under the control of the *MET*3 promoter, utilizing the *URA3* marker. A 3xHA tag was introduced at the C-terminus of Cek1 in wild type and *gem1*Δ*/*Δ strains by homologous recombination of the 3HA-*HIS1* cassette from the plasmid pFA-HA-HIS1 (Lavoie et al., 2008). Primers used in this study are listed in Table S1. Strains were grown at 30°C, 200 rpm unless otherwise indicated. Rich media was YPD (1% yeast extract, 2% peptone, 2% glucose) supplemented with 80 μg/mL uridine. Synthetic media was made with 0.67% w/v yeast nitrogen base without amino acids or carbohydrate and with ammonium sulfate (US Biological Y2025) with 80 μg/mL uridine and the appropriate amino acids. Repressive conditions for the ERMES mutants *mmm1*↓, *mdm10*↓ and *mdm12*↓ were achieved by addition of 2.5 mM methionine and 0.5 mM cysteine to the media.

**Table 1:** *C. albicans* strains used in this study.

**Strain**	**Genotype**	**References**	**Traven collection reference**
BWP17	ura3Δ::λimm434/ura3Δ::λimm434 arg4Δ::hisG/arg4Δ::hisG his1Δ::hisG/his1Δ::hisG	Wilson et al., 1999	YCAT15
DAY185	ura3Δ::λimm434/ura3Δ::λimm434 arg4Δ::hisG/ARG4::URA3::arg4Δ::hisG his1Δ::hisG/his1Δ::hisG::pHIS1	Davis et al., 2000	YCAT504
SN250	ura3Δ::λimm434::URA3- IRO1/ura3Δ::λimm434 arg4::hisG/arg4::hisG his1::hisG/his1::hisG leu2::hisG::CdHIS1/leu2::hisG::CmLEU2	Noble et al., 2010	YCAT639
*gem1Δ/Δ*	ura3Δ::λ imm434/ura3Δ::λ imm434 arg4Δ::hisG/arg4Δ::hisG his1Δ::hisG/hisΔ::hisG::pHIS1 gem1Δ::URA3/gem1Δ::ARG4	This study	YCAT679
*gem1Δ/Δ + GEM1*	ura3Δ::λ imm434/ura3Δ::λ imm434 arg4Δ::hisG/arg4Δ::hisG his1Δ::hisG/hisΔ::hisG::pHIS1-GEM1 gem1Δ::URA3/gem1Δ::ARG4	This study	YCAT673
*nuo1Δ/Δ*	ura3Δ::λimm434::URA3- IRO1/ura3Δ::λimm434 arg4::hisG/arg4::hisG his1::hisG/his1::hisG leu2::hisG/nuo11::CdHIS1/nuo1::CmLEU2	Noble et al., 2010	YCAT489
*mkc1*	ura3Δ::λimm434/ura3Δ::λimm434 arg4Δ::hisG/arg4Δ::hisG his1Δ::hisG/his1Δ::hisG mkc1::Tn7- UAU1/mkc1::Tn7-URA3	Blankenship et al., 2010	YCAT330
*cek1Δ/Δ*	ura3Δ::λ imm434/ura3Δ::λ imm434 arg4Δ::hisG/arg4Δ::hisG his1Δ::hisG/hisΔ::hisG::pHIS1 cek1Δ::URA3/cek1Δ::ARG4	This study	YCAT362
*CEK1- HA*	ura3Δ::λimm434/ura3Δ::λimm434 arg4Δ::hisG/ARG4::URA3::arg4Δ::hisG his1Δ::hisG/his1Δ::hisG CEK1-3xHA HIS1	This study	YCAT384
*gem1*Δ/Δ *CEK1-HA*	ura3Δ::λ imm434/ura3Δ::λ imm434 arg4Δ::hisG/arg4Δ::hisG his1Δ::hisG/hisΔ::hisG gem1Δ::URA3/gem1Δ::ARG4 CEK1- 3xHA HIS1	This study	YCAT819
*mmm1*↓	ura3Δ::λimm434/ura3Δ::λimm434 arg4Δ::hisG/arg4Δ::hisG his1Δ::hisG/his1Δ::hisG::pHIS1 mmm1Δ::ARG4/URA3-P_MET3_-MMM1	Tucey et al., 2016	YCAT595
*mdm10*↓	ura3Δ::λimm434/ura3Δ::λimm434 arg4Δ::hisG/arg4Δ::hisG his1Δ::hisG/his1Δ::hisG::pHIS1 mdm10Δ::ARG4/URA3-P_MET3_-MDM10	Tucey et al., 2016	YCAT597
*mdm12*↓	ura3Δ::λimm434/ura3Δ::λimm434 arg4Δ::hisG/arg4Δ::hisG his1Δ::hisG/his1Δ::hisG::pHIS1 mdm12Δ::ARG4/URA3-P_MET3_-MDM12	This study	YCAT599
*mmm1*↓+*MMM1*	ura3Δ::λimm434/ura3Δ::λimm434 arg4Δ::hisG/arg4Δ::hisG his1Δ::hisG/hisΔ::hisG::pHIS1-MMM1 mmm1Δ::ARG4/URA3-P_MET3_-MMM1	Tucey et al., 2016	YCAT697
*mdm12*↓*+MDM12*	ura3Δ::λimm434/ura3Δ::λimm434 arg4Δ::hisG/arg4Δ::hisG his1Δ::hisG/hisΔ::hisG::pHIS1-MDM12 mmm1Δ::ARG4/URA3-P_MET3_-MDM12	This study	YCAT693

For analysis of sensitivities to various drugs and chemicals, ten-fold serial dilutions of cultures starting form an OD_600_ = 0.5 were dropped on control plates, or plates containing the compounds indicated in the Figures. Plates were incubated at 30°C for 2 days and then photographed.

For testing filamentation on plates, *C. albicans* strains were plated on Spider plates (1% nutrient broth, 1% D-mannitol, 2 g K_2_HPO_4_) or RPMI-1640 plates. Plates were incubated for up to 5 days at 37°C or 30°C, and colonies examined and photographed with a stereo dissecting microscope (Olympus SZX 16 and Olympus MVX10).

For assaying filamentous growth in liquid media, cells were inoculated to an OD_600_ = 0.3 into pre-warmed YPD+10% FBS, Spider media or RPMI and incubated at 37°C for the times indicated in the figure legends.

### Antifungal susceptibility tests

MICs were determined using the broth microdilution method according to CLSI guidelines M27-A3. Drug concentrations ranged from 0.004 to 2 μg/ml for caspofungin; One-hundred-microliter portions of 2-fold serial dilutions of the drugs prepared in YPD were added into wells of 96-well plates. Overnight cultures were diluted to a density of 1 × 10^3^ to 5 × 10^3^ CFU/ml, and 100 μl was added to each well of a 96-well plate. Plates were incubated for 48 h at 30°C (the longer incubation time was additionally used to compensate for slow growth of the *gem1*Δ*/*Δ mutant strain). The MIC was defined as the concentration resulting in an inhibition of at least 50% of fungal growth.

### Quantitative PCR

Growth conditions and experimental set up are described in the figure legends. Total RNA was obtained by extraction with hot acid phenol. DNase treatment of 10 μg of RNA using TURBO DNase (Ambion) was performed according to the manufacturer's instructions. One μg of DNase-treated RNA was reverse-transcribed using SuperScript III Reverse Transcriptase (Invitrogen) according to the manufacturer's instructions. Quantitative PCR was done on LightCycler 480 (Roche) using the FastStart Universal SYBR Green Master Rox (Roche) reagent mix. The data was analyzed using the LinReg software (Ramakers et al., 2003; Ruijter et al., 2009). 18S RNA or *SCR1* served as control genes for normalization. Statistical analysis of qPCR data was performed using 2-way-ANOVA analysis with multiple comparison, and Tukey's multiple comparison test.

### Analysis of cell wall stress pathway activation by western blots

For assaying cell wall stress pathway activation, overnight cultures of yeast strains were diluted to an OD_600_ of 0.2 into fresh YPD and grown to early log phase, followed by treatment with 125 ng/ml caspofungin for the times indicated in the Figure. For ERMES shutdown strains *mmm1*↓, *mdm10*↓ and *mdm12*↓ a single colony was streaked in a patch on SD plates with all amino acids plus Met/Cys to achieve ERMES gene repression. The patch was taken up in PBS-buffer. Liquid SD medium containing all amino acids and Met/Cys was then inocculated to and OD_600_ of 0.2, and from there on treated as described above for the cultures grown in YPD. Whole-cell protein extracts were prepared by trichloroacetic acid (TCA) precipitation, and Western blotting was performed. Phospho-Mkc1 and phospho-Cek1 were detected using the rabbit monoclonal anti-phospho-p44/42 mitogen-activated protein (MAP) kinase antibody (Cell Signaling). Actin was detected with an anti-actin monoclonal antibody (Millipore). Cek1-HA (C-terminally tagged) was detected with the anti-HA antibody (Biolegend). Quantification of Western blot data for the phospho-Cek1 and actin bands in **Figure 3B** (from data shown in **Figure 3A** and Figure S3) was performed with 16-bit grayscale pictures of scanned films using the Toolbox module of ImageQuant 1D version 7.0. Background signals were subtracted using the local median method performed by the software. Chemiluminescent signals for protein levels of Cek1-3xHA (**Figures 3D,E**) were captured with the Biorad ChemiDoc^TM^ Touch Imaging System, and quantification of Cek1-3xHA and actin bands was performed using the Biorad Image Lab^TM^ software Version 6.0.0 build 26.

### Microscopy

All cell imaging was performed using an Olympus BX60 microscope equipped with Olympus with Spot Advanced Software, with bright field or fluorescent settings. Mitochondrial staining was performed with 1 μM MitoTracker Red CMXRos (Life Technologies, M7512), with the stock diluted into the growth media to give a final concentration of 0.1 μM, and then used as per the manufacturer's instructions. Images were taken with a 100 × objective, 72 DPI resolution (1,600 × 1,200 pixels) using an Olympus BX60 fluorescence microscope equipped with Spot Advanced Software.

Calcofluor white staining (250 μg/ml) was performed at room temperature in the dark for 10 min, followed by three washes in distilled water.

Immunofluorescence was performed after induction of hyphal formation in Spider media at 37°C for 3h. Cells were fixed with 3.7% formaldehyde (final concentration). Blocking with BSA was carried out for 1 h at room temperature. Primary anti-1, 3 beta glucan antibody (Biosupplies) incubation was carried out for 1 h (1:500 dilution), and secondary antibody anti-mouse Alexa Fluor488 (Invitrogen) incubation was carried out for 45min at room temperature (1:800 dilution). Antibodies were diluted in 3% BSA in PBS.

### Phospholipid analysis

Cultures were grown in YPD until mid-log phase and 20 OD units of each strain were harvested and washed three times with H_2_O. Cells were then resuspended in 400 μl of methanol and disrupted by vortexing with 100 μl of glass beads. Then 800 μl of chloroform was added and samples were vortexed and centrifuged to remove insoluble material. The organic supernatant was removed and washed once with 0.9% NaCl. The lipid containing phase was collected and dried under the stream of nitrogen. Lipids were separated by thin layer chromatography (TLC) on Slica 60 plates (Millipore) in chloroform/ethanol/water/trimethylamine (30:35:7:35). Dried TLC plates were stained with 470 mM CuSO_4_ in 8% o-phosphoric acid and incubated at 180°C for 15 min. Lipids standards (Avanti Polar Lipids) were used to identify cardiolipin (CL), phosphatidylcholine (PC), phosphatidylethanolamine (PE), and phosphatidylserine (PS). Phospholipids were quantified using FIJI software (version 2.0.0-rc43/1,5k).

### Worm infection assays

The worm-*C. albicans* infection assay was performed as described previously (Uwamahoro et al., 2012). Briefly, young adult nematodes were allowed to feed for 4 h on lawns of *C. albicans* wild type, *gem1*Δ/Δ or *gem1*Δ/Δ+*GEM1* grown on solid BHI media (DIFCO) + kanamycin (50 μg/ml). Worms were washed with M9 media and transferred into wells of a six-well microtiter dish (Corning) containing 2 ml of liquid media (80% M9 and 20% BHI) at 60 to 80 worms per well. The plates were incubated at 25°C, and worms were assessed at 24 h intervals for *C. albicans* filamentation using a DIC microscope and photographed using an Olympus IX81 microscope with the Olympus cellSense software. Worms were scored as follows: alive (no penetrating hyphae visible), dead with hyphae penetrating, and dead without penetrative hyphae (i.e., death caused by other reason unrelated to hyphal formation). Only 13 out of 1,925 worms (i.e., ≈0.67%) died without filamentation across the three experiments. In order to focus on the filamentation-induced host death, these 13 animals were excluded from the analyses. The percentage of dead worms with penetrative filamentation was determined from three independent experiments after 72 h of infection. Means and the standard deviation were calculated.

## Results

### *C. albicans* Gem1 controls mitochondrial morphology and fitness in the absence of respiratory growth functions or major lipid homeostasis defects

**Figure 1:**
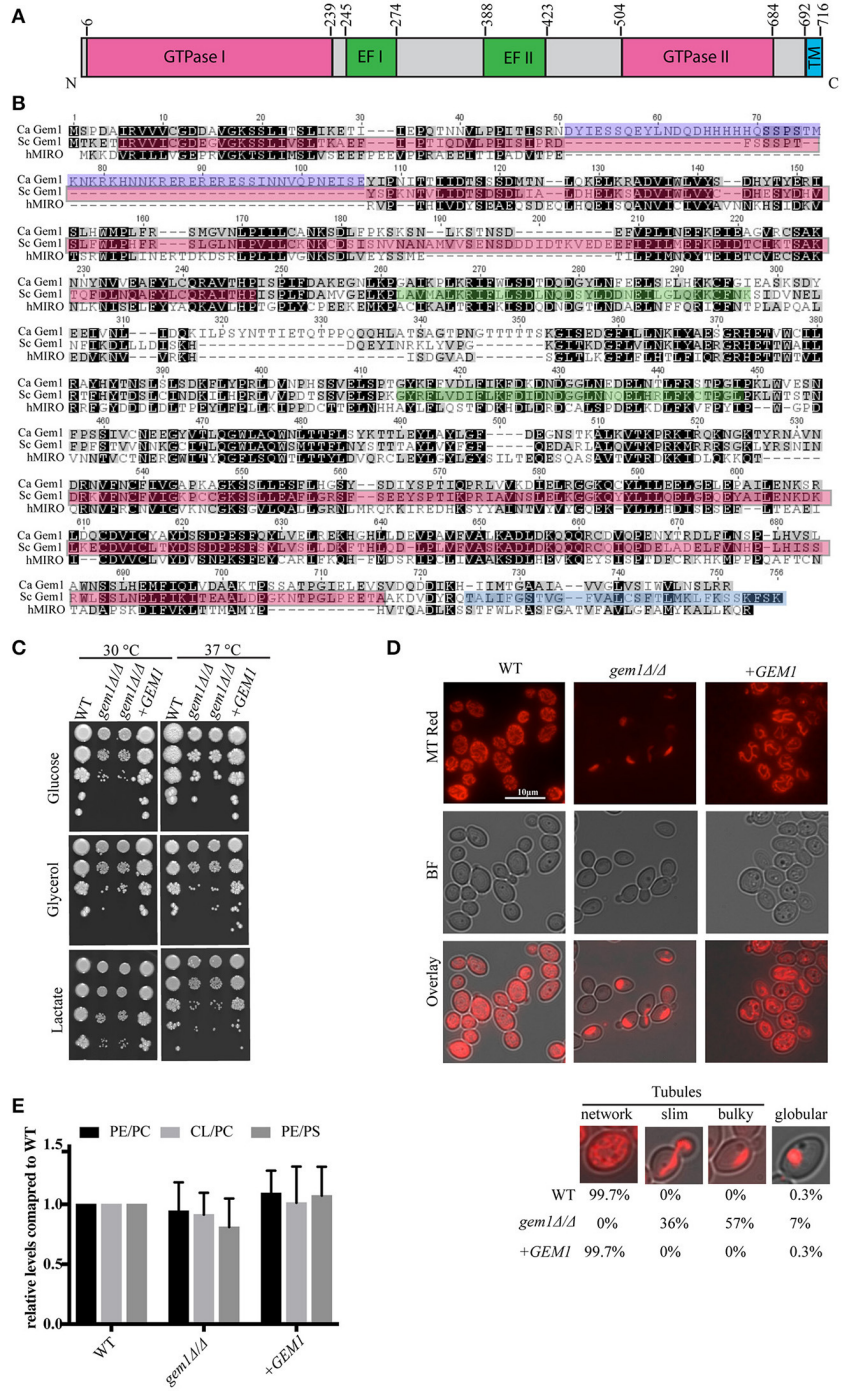
The dominant cellular function of *C. albicans* Gem1 is to maintain tubular mitochondrial morphology. **(A)** Domain structure of *C. albicans* Gem1. TM, Transmembrane domain; EF, Ca2^+^ binding EF hand domain. **(B)** Protein sequence alignment of Gem1 from *C. albicans, S. cerevisiae*, and their human ortholog MIRO1 (hMIRO). The colored regions represent the following: Red, GTPase I and II; violet, 56 amino acids not present in hMIRO or Sc Gem1; green, Ca^2+^ binding domain EF I and II; blue, transmembrane domain. The alignment was performed with MUSCLE MUltiple Sequence Comparison by Log-Expectation v3.8.31. **(C)** Growth of *C. albicans* wild type, two independent *gem1*Δ*/*Δ mutants and the complemented +*GEM1* strains on plates containing glucose, glycerol or lactate as the carbon source. Ten-fold serial dilutions were spotted, and plates and were photographed after 2 days of growth at the indicated temperatures. **(D)** Mitochondrial morphology was assessed after staining of cells with MitoTracker Red. MT, MitoTracker; BF, Bright field. The brightness of the fluorescent pictures was increased by 20% to create the overlay pictures. Mitochondrial phenotypes were quantified according to their appearance, assessing 600 cells per strain. **(E)** Phospholipids were separated by one-dimensional TLC and quantified as described in section Materials and Methods. A representative TLC is shown in Figure S2. PS, phosphatidylserine; PE, phosphatidylethanolamine; CL, cardiolipin; PC, phosphatidylcholine. Shown are the mean and standard deviation from three biological replicates.

The *C. albicans* Gem1 is encoded by C1_00890W (orf19.6016), with characteristic domain structure consisting of GTPase domains I and II, Ca^2+^ binding domains I and II and a transmembrane domain (Figure 1A). Interestingly, a protein sequence alignment uncovered a stretch of 56 amino acids located in the GTPase Domain I that is unique to *C. albicans* Gem1, and not found in either *S. cerevisiae* Gem1 nor human Miro1 or Miro2 (Figure 1B and Figure S1). A BLAST search of this stretch of amino acids revealed that, in addition to *C. albicans*, it exists in only one other organism, the closely related species *Candida dubliniensis*. It is not clear what impact this stretch has on the structure or activity of GTPase Domain I in *C. albicans*, but we do anticipate the GTPase Domain I to function normally, as the predicted GTP-binding site, the G1 motif, is highly conserved in the *C. albicans* Gem1 protein. The *C. albicans* homozygous deletion mutant *gem1*Δ*/*Δ showed reduced growth on rich glucose media at 30°C, but this growth defect was not exacerbated at 37°C or in media containing the non-fermentable carbon sources glycerol or lactate (Figure 1C). Two independent *gem1*Δ*/*Δ deletion clones showed comparable phenotypes, and we could restore fitness to the mutant by re-introduction of the wild type *GEM1* gene (Figure 1C). In the *gem1*Δ*/*Δ mutant the tubular mitochondrial network structure was lost, and collapsed tubules and some mitochondrial globular spheres were evident (Figure 1D). Occasionally, we noticed some cells with no detectable mitochondrial staining, however this was comparable between the wild type and *gem1*Δ*/*Δ mutant strains and occurred in 1–5% of the cell population. Unlike the drastic difference in mitochondrial morphology between the wild type and the *gem1*Δ*/*Δ mutant, no differences in steady state phospholipid levels were observed in the mutant compared to the wild type strain (Figure 1E).

### Gem1 regulates cell surface integrity and the Cek1-dependent cell wall stress response

**Figure 2:**
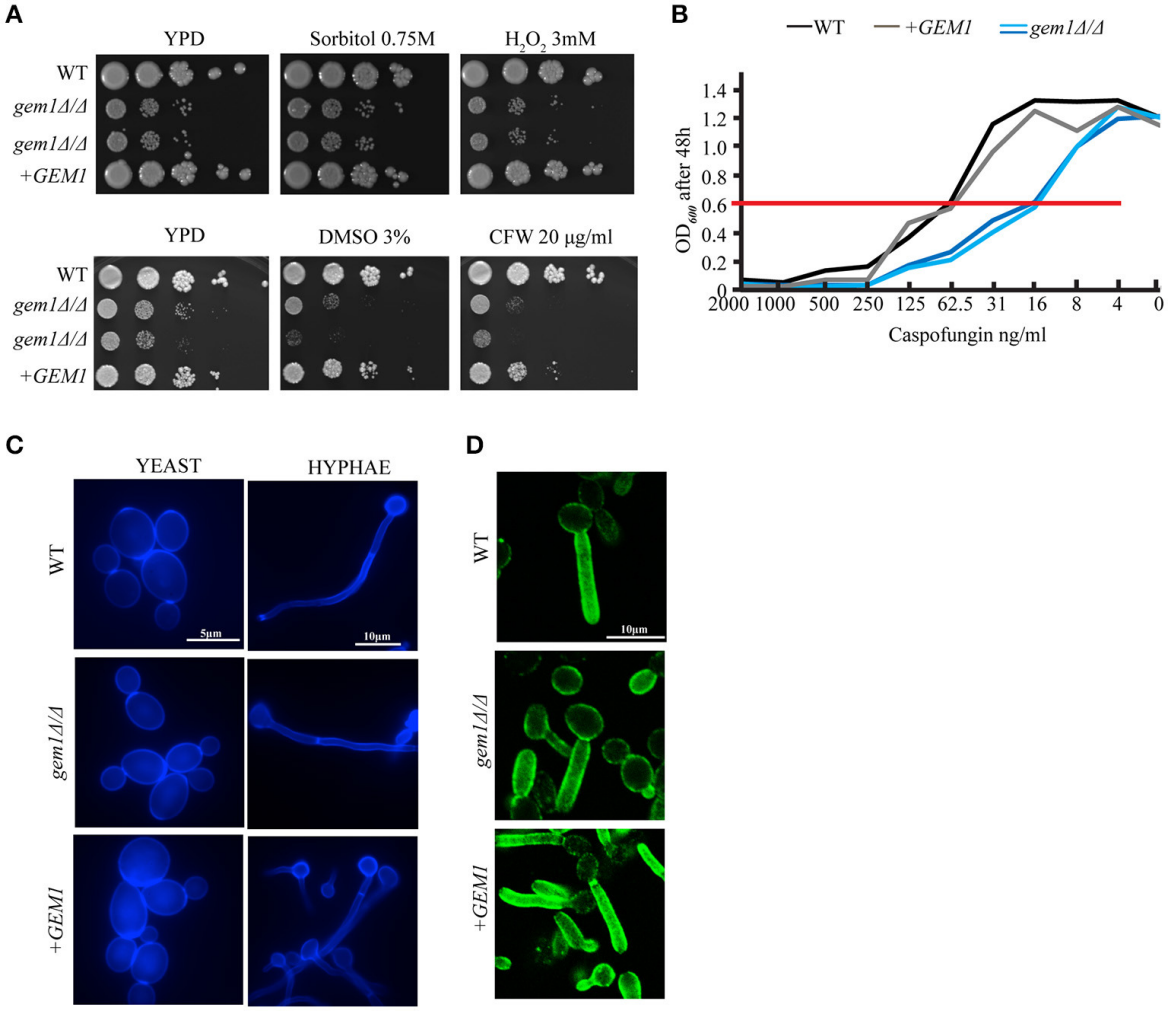
Gem1 controls susceptibility to cell wall stress. **(A)** Growth of *C. albicans* wild type, *gem1*Δ*/*Δ mutants and the complemented strain (+*GEM1*) was tested on YPD plates in the presence of the indicated drugs at 30°C. Ten-fold serial dilutions were spotted and plates were photographed after 2 days. (CFW, Calcofluor White). **(B)** Determination of caspofungin minimal inhibitory concentrations for wild type *C. albicans*, two independent *gem1*Δ*/*Δ mutants and the complemented strain (+*GEM1*). OD_600_ was measured after 48 h of growth in YPD media at 30°C and plotted against the caspofungin concentrations used. The red line indicates the MIC_50_. Three independent experiments were performed, and gave the same result for the MICs. Shown is one representative experiment. **(C)** Calcofluor white staining of yeast and hyphae. Hyphal formation was induced for 3 h in Spider medium at 37°C. **(D)** Immunofluorescence analysis of formaldehyde fixed cells was performed with the anti-β (1,3)-glucan antibody. Hyphae were induced in Spider media at 37°C for 3 h.

**Figure 3:**
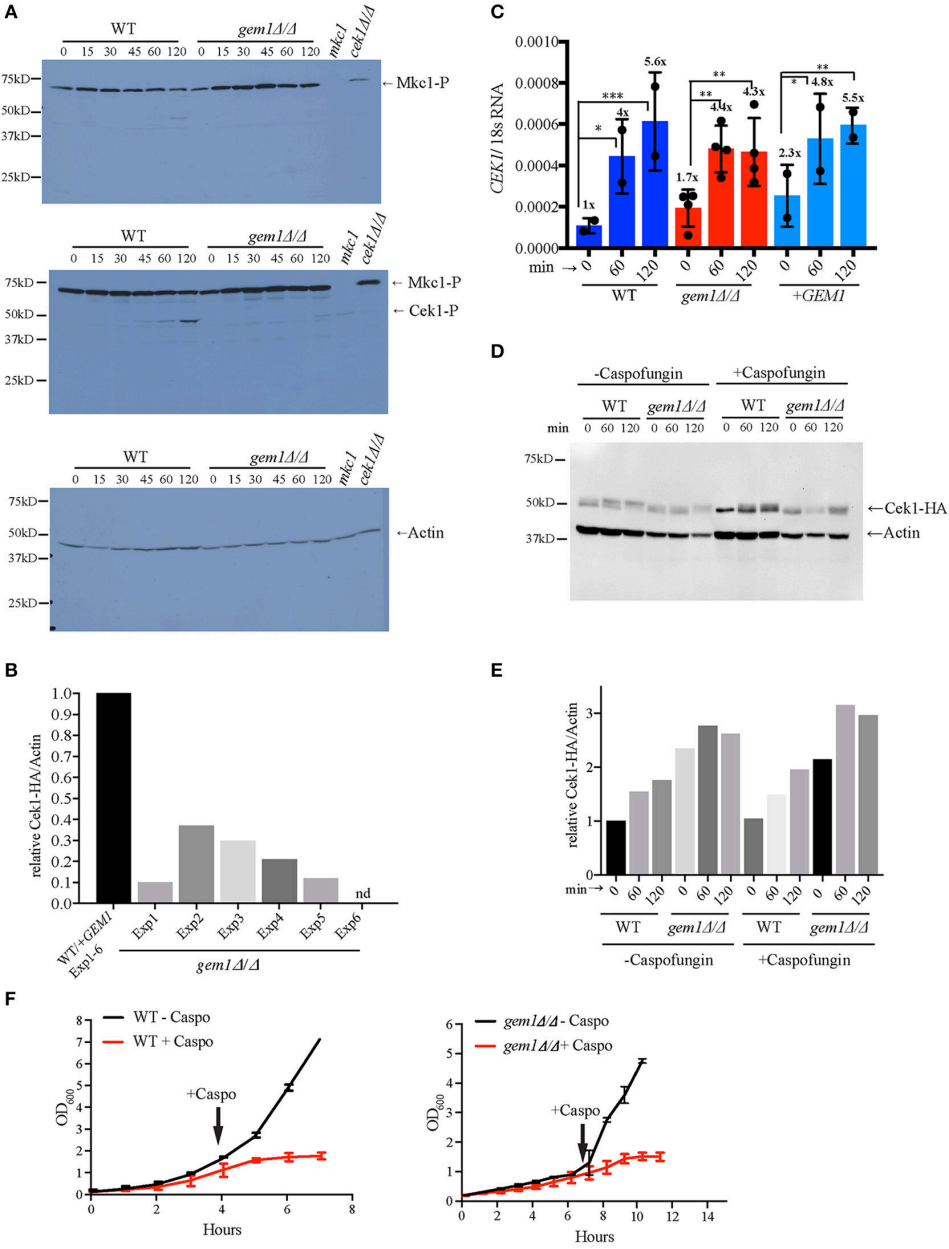
Gem1 is required for activation of the Cek1-dependent cell wall stress pathway. **(A)** Cells were grown in YPD to early log phase and then treated with 125 ng/ml caspofungin for the indicated times. The *mkc1* and *cek1* mutant samples are from cultures treated with caspofungin for 2 h. Phospho-Mkc1 and phospho-Cek1 were detected using the anti-phospho Erk antibody (p-44/42). The top and middle panel are from the same membrane—top: short exposure to avoid saturation of the phospho-Mkc1 signal; middle: longer exposure of the same membrane to obtain the phospho-Cek1 signal. Actin was used as the loading control. **(B)** The phospho-Cek1 signal from wild type and *gem1*Δ*/*Δ mutant and was quantified at 120 min post caspofungin treatment, and normalized to the signal for actin as the loading control. Quantification was performed as described in the section Materials and Methods. The normalized phospho-Cek1/Act1 ratios in the mutant were expressed relative to the wild type, which was set to 1. Five independent experiments were analyzed. The primary data used for these quantifications are shown in Figure 3A and Figure S3. **(C)** Expression levels of *CEK1* mRNA were monitored by quantitative PCR at the indicated time points after caspofungin treatment. 18S RNA was used for normalization. Cells were grown as described in **(A)**. Shown are results from two independent experiments (average and standard deviation). In each of the experiments, two independent *gem1*Δ*/*Δ mutant clones were used (therefore, for the mutant *n* = 4). The numbers above the graph represent fold up-regulation relative to untreated wild type samples (which were set to 1). Statistical analysis was performed using 2-way-ANOVA with multiple comparison, followed by Tukey test. ^*^p > 0.05, ^**^p > 0.001, ^***^p > 0.0002. As shown in the Figure, the increase in *CEK1* transcription over time is statistically significant in both wild type and *gem1*Δ*/*Δ mutant strains, showing that both strains are able to trigger *CEK1* transcription in response to cell wall stress. Moreover, statistical analysis revealed no significant difference between the two strains (wild type and *gem1*Δ*/*Δ mutant) in triggering *CEK1* activation under these conditions. **(D)** Total protein levels of Cek1 were determined using HA-tagged Cek1 in wild type and *gem1*Δ*/*Δ strains. Actin was used as a loading control. Cells were grown as described in **(A)**. **(E)** Quantification of total levels of Cek1-3HA relative to the actin control, in the presence or absence of caspofungin treatment. The Cek1-3HA/actin ratio for the wild type strain (WT) in the absence of caspofungin at 0 min was set to 1, and all other values for the wild type and mutant were calculated relative to that. **(F)** Cells of the indicated strains were grown in YPD media as described in **(A)**. Caspofungin (125 ng/ml final concentration) was added at the indicated time points and growth was monitored by measuring optical density. Note that, since the *gem1*Δ*/*Δ mutant grows slower than the wild type, the time for the mutant to reach log phase and caspofungin addition was longer. Shown are the mean and standard deviation of three independent experiments.

**Figure 4:**
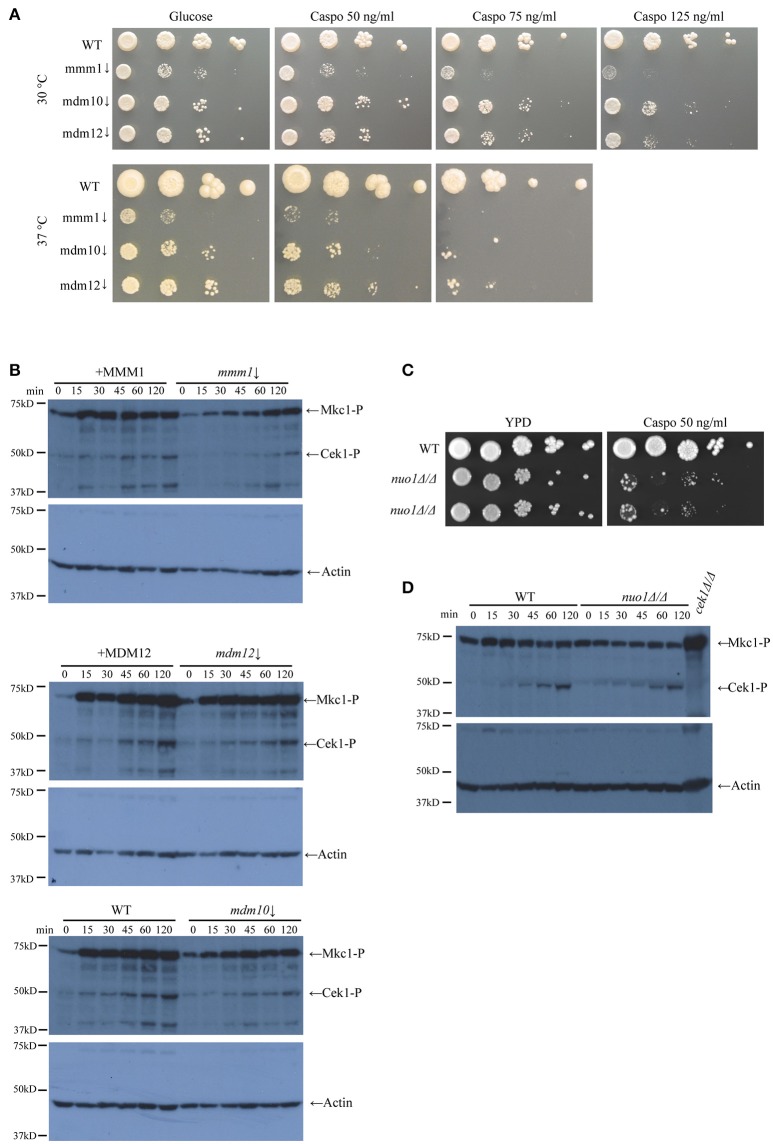
ERMES activity is needed for optimal growth of *C. albicans* upon cell wall stress. **(A)** Conditional mutants of ERMES (*mmm1*↓, *mdm1*0↓ *mdm12*↓) were grown in synthetic media+Met/Cys, in the presence of the indicated concentrations of caspofungin (Caspo). Ten-fold serial dilutions were spotted and pictures taken after 3 days of growth at the indicated temperatures. **(B)** The activation of Cek1 and Mkc1 was tested in the conditional ERMES complex mutants as described in Figure 3A. Strains were grown in synthetic medium with all amino acids and methionine and cysteine were added in to repress ERMES gene expression. As controls, either the wild type strain or the complemented strains were used, as indicated in the Figure. **(C)** Growth of wild type and *nuo1*Δ*/*Δ mutants was tested on YPD plates in the presence of 50 ng/ml caspofungin (Caspo). Ten-fold serial dilutions were spotted and plates were photographed after 2 days of growth at 30°C. **(D)** Activation of Mkc1 and Cek1 in *nuo1*Δ*/*Δ was tested as described in Figure 3A.

Mitochondria are centrally important to cellular physiology under normal conditions, and also in response to various stresses. We therefore tested the susceptibility of the *gem1*Δ*/*Δ mutant to a range of stresses that are relevant for *C. albicans* in a disease context. The growth defect of the *C. albicans gem1*Δ*/*Δ was not exacerbated by osmotic or oxidative stress (Figure 2A), indicating that the Gem1 does not participate in the response to these stressors. In contrast, the *gem1*Δ*/*Δ mutant was susceptible to cell wall stress caused by calcofluor white and the 1,3 ß-glucan synthase inhibitor caspofungin, and displayed susceptibility to the solvent DMSO that compromises membrane integrity (Figures 2A,B). Despite the susceptibility to cell wall stress, the *gem1*Δ*/*Δ mutant displayed normal distribution of chitin (as judged by calcofluor white staining), and also normal distribution of 1, 3 ß-glucan (as judged using immunofluorescence with the anti-1, 3 ß-glucan antibody) (Figures 2C,D). Therefore, we next assessed whether the mutant was able to activate the cell wall stress pathways in response to caspofungin-inflicted cell wall damage. For this, we used Western blots to monitor the appearance of the phosphorylated form of the kinase Mkc1 (which reports on the activation of the Cell Wall Integrity (CWI) pathway), and the appearance of the phosphorylated form of the kinase Cek1 (which reports on the activation of the Cek1-dependent pathway). Mutant strains of *MKC1* and *CEK1* were used to ascertain the identities of the phospho-Cek1 and phospho-Mkc1 bands (Figure 3A). The *gem1*Δ*/*Δ mutant was able to trigger activation of the CWI pathway normally (Figure 3A). However, unlike in the wild type strain, where the phospho-Mkc1 signal declined over time as cells repaired the damage to their cell wall and therefore turned the pathway off, Mkc1 remained phosphorylated for a prolonged time in the *gem1*Δ*/*Δ mutant, consistent with reduced ability of the mutant to repair cell wall damage (Figure 3A). In contrast to prompt activation of the CWI pathway, activation of the Cek1 pathway was delayed in the *gem1*Δ*/*Δ mutant (Figure 3A). Although Western blots are semi-quantitative, to gage the extent of the defect in the appearance of the phospho-Cek1 band in the *gem1*Δ*/*Δ mutant, we quantified the phospho-Cek1 signal at 120 min after caspofungin treatment relative to the actin loading control (the primary data used in these quantifications is shown in Figure 3A and Figure S3. Note that five out of six experiments were used for quantification due to fact that in experiment 6 the phospho-Cek1 signal in the mutant displayed no detectable intensity levels during quantification). This analysis showed a consistent reduction of phospho-Cek1 in the *gem1*Δ*/*Δ mutant compared to the wild type (Figure 3B). The *gem1*Δ*/*Δ mutant was able to up-regulate transcription of the *CEK1* gene following caspofungin treatment (Figure 3C), and the total protein levels of Cek1 in the *gem1*Δ*/*Δ mutant were comparable to the wild type strain in the presence or absence of caspofungin treatment (Figures 3D,E; HA-tagged Cek1 was used in these experiments to detect total Cek1 protein levels). Therefore, low levels of Cek1 pathway activation in the *gem1*Δ*/*Δ mutant are likely due to a signaling defect, rather than low expression levels of Cek1. Cek1 is known to be phosphorylated in response to re-entry of cells into active growth (Román et al., 2009a). Given that the *gem1*Δ*/*Δ mutant is hyper susceptible to caspofungin, we wondered whether the delayed phosphorylation of Cek1 was caused by slower resumption of growth after cell wall stress. However, growth assays did not support this hypothesis: in both wild type and *gem1*Δ*/*Δ mutant cultures, caspofungin treatment caused a growth arrest that lasted for several hours (Figure 3F). Yet, the wild type strain was triggering Cek1 phosphorylation during the prolonged growth arrest (i.e., despite lack of growth in caspofungin-containing media), while the *gem1*Δ*/*Δ mutant was not able to activate Cek1 for a prolonged period of time (Figure 3A). Mutants in the ERMES complex also displayed caspofungin susceptibility, which was even more pronounced at 37°C compared to 30°C (Figure 4A). We note that ERMES genes are near essential in *C. albicans*, and therefore we used conditional mutants in which the expression of the ERMES genes is driven by the *MET3* promoter and is therefore repressible by methionine and cysteine (Tucey et al., 2016). ERMES mutants were generally able to promptly trigger activation of the CWI pathway in response to cell wall stress (see phospho-Mkc1 Western blots in Figure 4B), although somewhat reduced levels of phospho-Mkc1 were seen in the *mmm1* and *mdm10* mutants at the early time points (Figure 4B). Cek1 pathway activation in response to caspofungin was clearly retarded in the *mmm1* mutant, while the *mdm10* and *mmm12* mutants did not display a major defect (Figure 4B). We next assessed how inhibition of mitochondrial respiratory chain activity would affect cell wall stress pathway activation. To this end, we used a mutant in the mitochondrial respiratory complex I (*nuo1*Δ*/*Δ), as complex I activity has been shown to be required for cell wall integrity in *C. albicans* (She et al., 2013, 2015, 2016). The *nuo1*Δ*/*Δ was susceptible to caspofungin (Figure 4C), but activated Cek1 normally in response to caspofungin treatment (Figure 4D).

### Gem1 controls invasive hyphal growth of *C. albicans*

**Figure 5:**
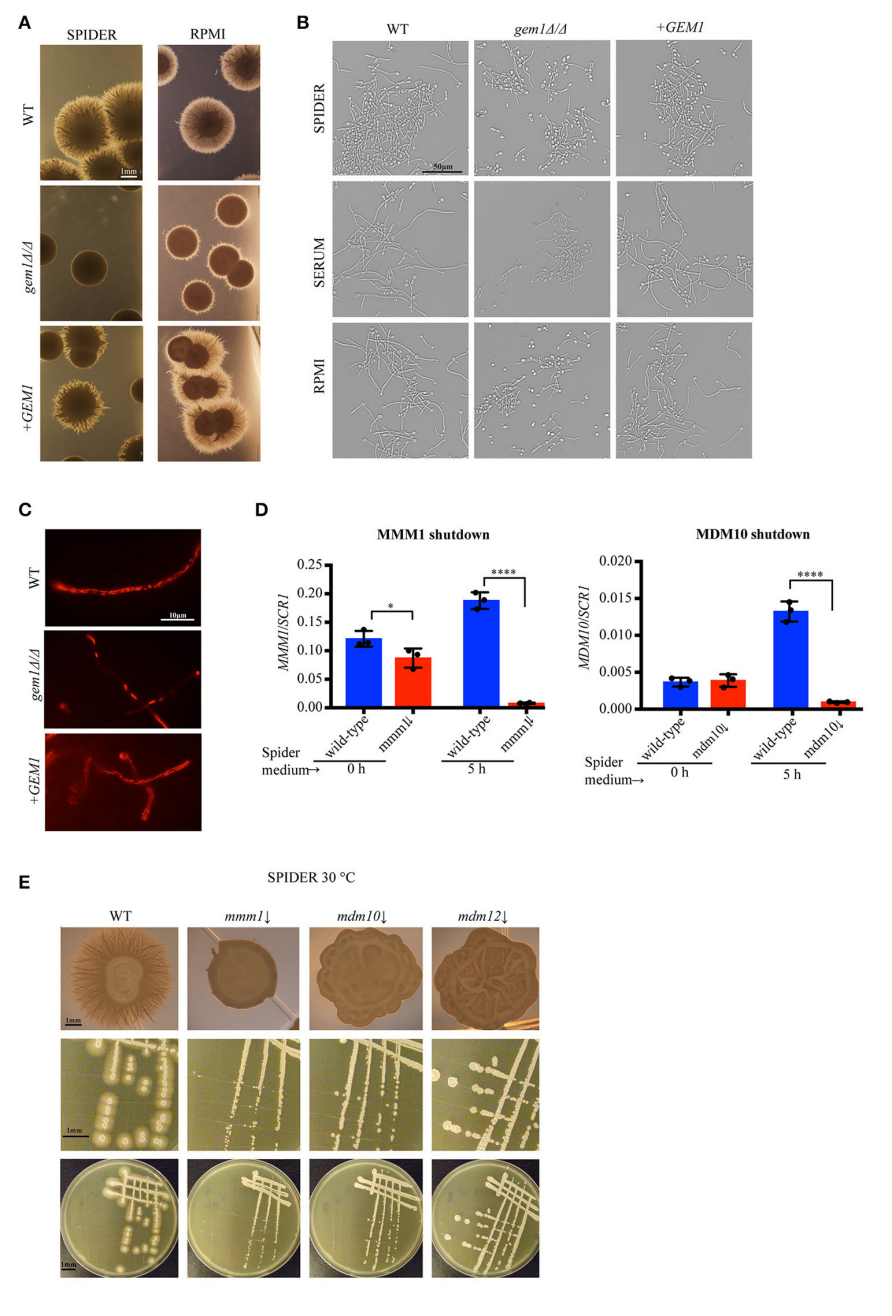
Gem1 and ERMES function in invasive growth of *C. albicans*. **(A)** Filamentous growth of wild type (WT), *gem1*Δ*/*Δ and the complemented +*GEM1* strain was tested on Spider and RPMI plates at 37°C. Pictures were taken after 5 days. **(B)** Filamentous growth of wild type (WT), *gem1*Δ*/*Δ and the complemented +*GEM1* strain was tested in liquid Spider, YPD +10% Serum and RPMI liquid media. Pictures were taken after 3 h of incubation at 37°C. **(C)** Mitochondria of wild type (WT), *gem1*Δ*/*Δ and the complemented +*GEM1* strain were stained with MitoTracker Red after incubation for 3 h in Spider media. **(D)** Efficient repression of ERMES gene expression in the *mmm1*↓ *and mdm10*↓ strains in Spider media was confirmed by quantitative PCR. The mean and standard deviation of three independent experiments is shown. Statistical significance was determined by unpaired *t*-test with Welch's correction^*^p < 0.03, ^****^p < 0.0001. **(E)** ERMES mutant strains *mmm1*↓*, mdm10*↓ and *mdm12*↓ were grown on Spider plates and colony morphology was observed after incubation at 30°C for 5 days. Top row: single colony morphology of the indicated strains, middle row: close-up of selected areas of the plates (bottom row).

The capacity for invasive hyphal growth is important for pathogenicity of *C. albicans*, due to roles such as tissue invasion and evasion of innate immune responses (Sudbery, 2011; Jacobsen and Hube, 2017). Hyphal growth of the *gem1*Δ*/*Δ mutant was drastically reduced on mannitol-containing solid Spider media (Figure 5A). Hyphal outgrowth from the colonies was only sparsely observed, and the hyphae were much shorter than those emanating from wild type colonies, even after prolonged growth (Figure 5A). A similar hyphal defect was seen on RPMI plates (Figure 5A). Unlike on solid plates, the *gem1*Δ*/*Δ mutant was able to produce hyphae in liquid Spider, YPD + serum and RPMI media, although fewer cells underwent the transition from yeast to hyphae and in some cases shorter hyphae were observed in the mutant possibly due to slower growth (Figure 5B). Mitochondrial morphology in the hyphal cells of the *gem1*Δ*/*Δ mutant was consistent with what we observed in yeast cells, in that large collapsed mitochondria were seen, and they were distributed along the length of the filaments showing they can traffic between hyphal cells (segments) (Figure 5C). We have previously reported that the ERMES mutant *mmm1* is able to filament in liquid RPMI media (Tucey et al., 2016). Given the specific defect of the *gem1*Δ*/*Δ mutant in filamentation on a solid substrate (i.e., plates), and a very similar mitochondrial morphology defect in hyphal cells of the *gem1*Δ*/*Δ mutant (Figure 5C) and the *mmm1* mutant (Tucey et al., 2016), we wondered whether, similarly to Gem1, ERMES is also needed for filamentation on solid substrates. Due to a growth defect of ERMES strains at 37°C (Tucey et al., 2016), we performed the experiment at 30°C on Spider plates to separate general growth defects from reduced hyphal morphogenesis. We also confirmed that Spider media was repressive for the *MET3* promoter driving ERMES gene expression in the conditional mutants (Figure 5D). As shown in Figure 5E (top panel), same as the *gem1*Δ*/*Δ mutant, the ERMES mutants *mmm1, mdm10* and *mdm12* were unable to produce the penetrative filaments surrounding the colonies on Spider plates. The colonies of the ERMES mutants grew well under these conditions: the *mdm12* mutant grew as well as the wild type, and the *mmm1* and *mdm10* mutants displayed reduced growth, but were very clearly growing substantially. This shows that ERMES function in hyphal invasion on agar plates can be uncoupled from growth defects (Figure 5E, middle and bottom panel).

**Figure 6:**
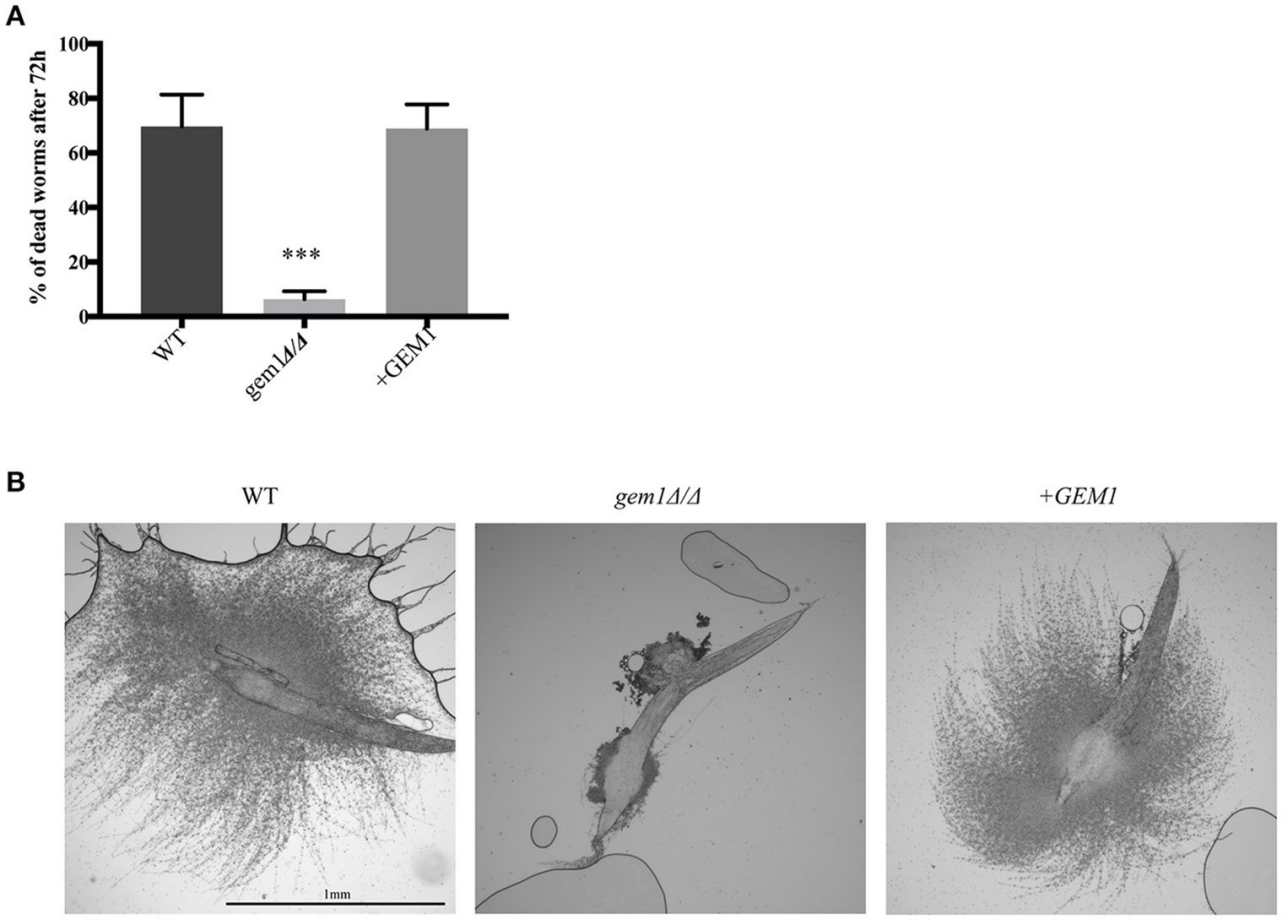
Gem1 is required for virulence of *C. albicans*. **(A)**. The worm *C*. *elegans* was infected with wild type, *gem1*Δ*/*Δ or the complemented +*GEM1* strain, and dead worms counted after 72 h. The mean and standard deviation of three independent experiments is shown. Statistical significance was determined by unpaired *t*-test with Welch's correction^***^p < 0.005. **(B)** Penetrative filamentation was monitored and pictures were taken after 72 h.

Unlike on plates, where hyphae invade into the agar, there is no resistance to hyphal growth in liquid media. Therefore, we interpret our hyphal morphogenesis results to mean that Gem1 and ERMES have roles in invasive hyphal growth, rather than hyphal morphogenesis *per se*. To further corroborate this proposition we used the worm infection model. The worm model relies on hyphal formation by *C. albicans* to pierce through the cuticle of the worm to kill it. This model has been used successfully to define *C. albicans* genes required for virulence, and it recapitulates virulence processes in mammalian infection models (Pukkila-Worley et al., 2009, 2011; Jain et al., 2013; Issi et al., 2017). As shown in Figure 6A, the *gem1*Δ*/*Δ mutant was drastically compromised in its ability to cause death of the worm host by hyphal penetration, with only 10–20% of the worms killed by the mutant 72 h following infection. Most worms infected by the *gem1*Δ*/*Δ mutant did not display any penetrative filamentation and survived the infection. We considered that the slow growth phenotype of the *gem1*Δ*/*Δ mutant might mean that a longer time is needed for penetrative filamentation to be observed. However, even at day 7 post infection, the worms infected with the mutant were mostly devoid of fungal filaments (data not shown). Accounting for the 10–20% of host killing in infection with the *gem1*Δ*/*Δ mutant (Figure 6A), occasionally hyphae were seen emanating from the worm (Figure 6B). However, these hyphae never achieved the length and numbers seen in infection with the control strains (Figure 6B).

## Discussion

Here we report the first characterization of the mitochondrial GTPase and ERMES subunit Gem1 in a pathogenic fungal species. Our study is consistent with the dominant role of Gem1 in *C. albicans* being to maintain tubular mitochondrial morphology. Firstly, in the absence of Gem1, extensive, interconnected mitochondrial tubules, such as seen in control strains, were lacking, and the predominant morphology was that of collapsed short tubules, and occasional globular mitochondria. Secondly, the *gem1*Δ*/*Δ mutant grew on non-fermentable carbon sources and is therefore capable of respiratory activity although the mitochondria are severely misshapen (in other words, mitochondrial morphology is compromised in the mutant, but respiration is not). Thirdly, steady state phospholipid levels were not different in the *gem1*Δ*/*Δ mutant relative to control strains. While this result does not exclude a role for Gem1 in ER-mitochondria phospholipid movements, as has been suggested based on data in *S. cerevisiae* (Kornmann et al., 2011), it does show that there is no major disruption of phospholipid homeostasis in the *C. albicans gem1*Δ*/*Δ strain under the conditions tested here. Finally, as shown during the highly polarized hyphal growth (Figure 5C), although they were misshapen, mitochondria were able to travel over large distances in the absence of Gem1 in *C. albicans*, which is consistent with Gem1 not being required for mitochondrial transport in *S. cerevisiae* (Frederick et al., 2004). Moreover, mitochondria were visible in all hyphal segments in the *gem1*Δ*/*Δ mutant, indicating that they can be inherited during cell division. Our previous work using conditional mutants of ERMES, and assaying the temporal appearance of mitochondrial, phospholipid and growth defects is consistent with the primary function of *C. albicans* ERMES also being in ensuring tubular mitochondrial morphology (Tucey et al., 2016). In fact, the mitochondrial morphology phenotype observed here for the *C. albicans gem1*Δ*/*Δ mutant is equivalent to what is seen early on upon inactivation of ERMES (Tucey et al., 2016): although in the ERMES mutants tubular mitochondria will eventually completely collapse into a globular structure, shortly upon conditional inactivation of ERMES there is an intermediate phase when a mixture of shorter collapsed tubules and globules is evident, closely matching the mitochondrial morphology phenotype shown here for the *gem1*Δ*/*Δ mutant. Unlike the ERMES genes, which are practically essential in *C. albicans*, particularly at 37°C and for using non-fermentable carbon sources (Tucey et al., 2016), *GEM1* is not an essential gene in this yeast pathogen, and it is not required for growth or viability at 37°C or while using non-fermentable carbon sources (Figure 1C). Collectively, our data are consistent with Gem1 and ERMES playing related roles in *C. albicans*, and suggest that in *C. albicans* both Gem1 and ERMES chiefly control mitochondrial morphology and dynamics. Our phenotypic analysis of the *gem1*Δ*/*Δ and ERMES mutants in *C. albicans* are further consistent with reports in *S. cerevisiae* of Gem1 being a regulatory, but not a structural or an essential subunit of ERMES (Kornmann et al., 2011; Nguyen et al., 2012). Future studies will need to directly address whether Gem1 physically interacts with the ERMES complex in *C. albicans*, and how Gem1 might regulate ERMES function in this fungal pathogen.

Our results further show that collapsed tubular network structure that occurs upon inactivation of Gem1 or ERMES has consequences for two crucial virulence traits of *C. albicans*: invasive hyphal growth and the response to cell wall stress. It is specifically the ability of hyphal cells to invade that is compromised in the absence of Gem1 or ERMES, not the ability to transition from yeast to hyphal growth. We conclude this based on the fact that both *gem1*Δ*/*Δ and the ERMES mutant *mmm1* can form hyphae in liquid media (as shown here for *gem1*Δ*/*Δ and in Tucey et al., 2016 for *mmm1*), but they defective in forming hyphae on solid media, where invasive growth is needed. We further used the worm infection model to show that penetration of *gem1*Δ*/*Δ mutant hyphae through tissues was almost completely absent. The ability of *C. albicans* to be a successful pathogen is multifaceted, and the worm infection model is predominantly focused on hyphal formation as a key virulence property. Nevertheless, given the slow growth phenotype of the *gem1*Δ*/*Δ mutant, its defect in forming hyphae in multiple conditions and the general requirement for mitochondrial functions for full virulence of *C. albicans* (Shingu-Vazquez and Traven, 2011; Calderone et al., 2015), it is likely that the *gem1*Δ*/*Δ mutant will be hypovirulent in a mammalian model as well. Mitochondrial respiration has been implicated in hyphal morphogenesis by regulating the Ras-cAMP pathway via ATP production (Grahl et al., 2015), or by maintaining NAD^+^ levels to drive NAD^+^ dehydrogenase activity needed for mannitol utilization in hyphae-inducing media such as Spider (Huang et al., 2017). The mechanism by which Gem1 controls hyphal invasion is different, because the *gem1*Δ*/*Δ mutants can grow on mannitol-containing Spider plates, and is not respiratory deficient. Instead, our data indicates that Gem1 controls the activation of the Cek1 kinase, which is part of a central mitogen activated protein (MAP) kinase pathway needed for both invasive hyphal growth of *C. albicans* and the response to cell wall stress (Csank et al., 1998; Li et al., 2009; Román et al., 2009b; Cantero and Ernst, 2011; van Wijlick et al., 2016), reviewed in Ernst and Pla (2011). Therefore, the inability of *C. albicans* to trigger prompt Cek1 activation in the absence of Gem1 could explain both the hypersusceptibility to cell wall stress, and the reduced hyphal invasion of the *gem1*Δ*/*Δ mutant. Interestingly, while the *gem1*Δ*/*Δ mutant had a very strong defect in Cek1 activation upon cell wall stress, the ERMES mutants had a more modest defect in triggering Cek1 pathway activation, with the *mmm1* mutant strain being the only one of the ERMES mutants to show a Cek1 activation delay. While the *gem1*Δ*/*Δ is a null strain with a homozygous deletion of *GEM1*, the ERMES mutants are conditional mutants, and it is therefore possible that some level of ERMES function is maintained in time frame post gene repression that was used in the cell wall stress experiments (18 h). The plate filamentation assays, for which the ERMES mutants showed compromised filamentation, are done over a prolonged period of several days of growth in repressive conditions. It is also possible that Gem1 has functions in Cek1 activation that are unrelated to ERMES.

Control of the Cek1 pathway in *C. albicans* is a novel mechanism of mitochondrial involvement in cell wall stress responses and invasive growth phenotypes. Our results show that specifically mitochondrial network morphology is needed for Cek1 activation, and not respiratory capacity of mitochondria, because the *gem1*Δ*/*Δ mutant is not respiratory deficient, and the mitochondrial complex I mutant *nuo1*Δ*/*Δ is able to trigger Cek1 phosphorylation in response to cell wall stress. An important question for future studies is to understand why disrupting mitochondrial morphology impairs Cek1 kinase activation. Some possibilities can be envisaged. Cek1 activity is controlled by plasma membrane localized sensors Msb2, Sho1 and Opy2 (Román et al., 2005, 2009b; Herrero de Dios et al., 2013). While the steady state phospholipid composition of membranes is not changed in the *gem1*Δ*/*Δ mutant, the mutant is susceptible to the organic solvent DMSO, suggesting that there are changes to membrane integrity in the mutant cells that could potentially impair the function and/or targeting of the membrane-localized sensors of the Cek1 pathway. In this context, it is interesting to note that there is a precedent for mitochondria playing a role in targeting of proteins to the plasma membrane (Wang and Deschenes, 2006). Thinking more generally about possible ways in which mitochondrial morphology might impair Cek1 pathway activation, mitochondria are known to “talk” to the plasma membrane via membrane-contact sites (Klecker et al., 2013; Lackner et al., 2013), reviewed in (Westermann, 2015), and changes to mitochondrial morphology, such as those found in the *gem1*Δ*/*Δ mutant, could perturb these contacts and thereby impair the sensing or transduction of the cell wall stress signal from the membrane to activate the Cek1 pathway.

**Figure 7:**
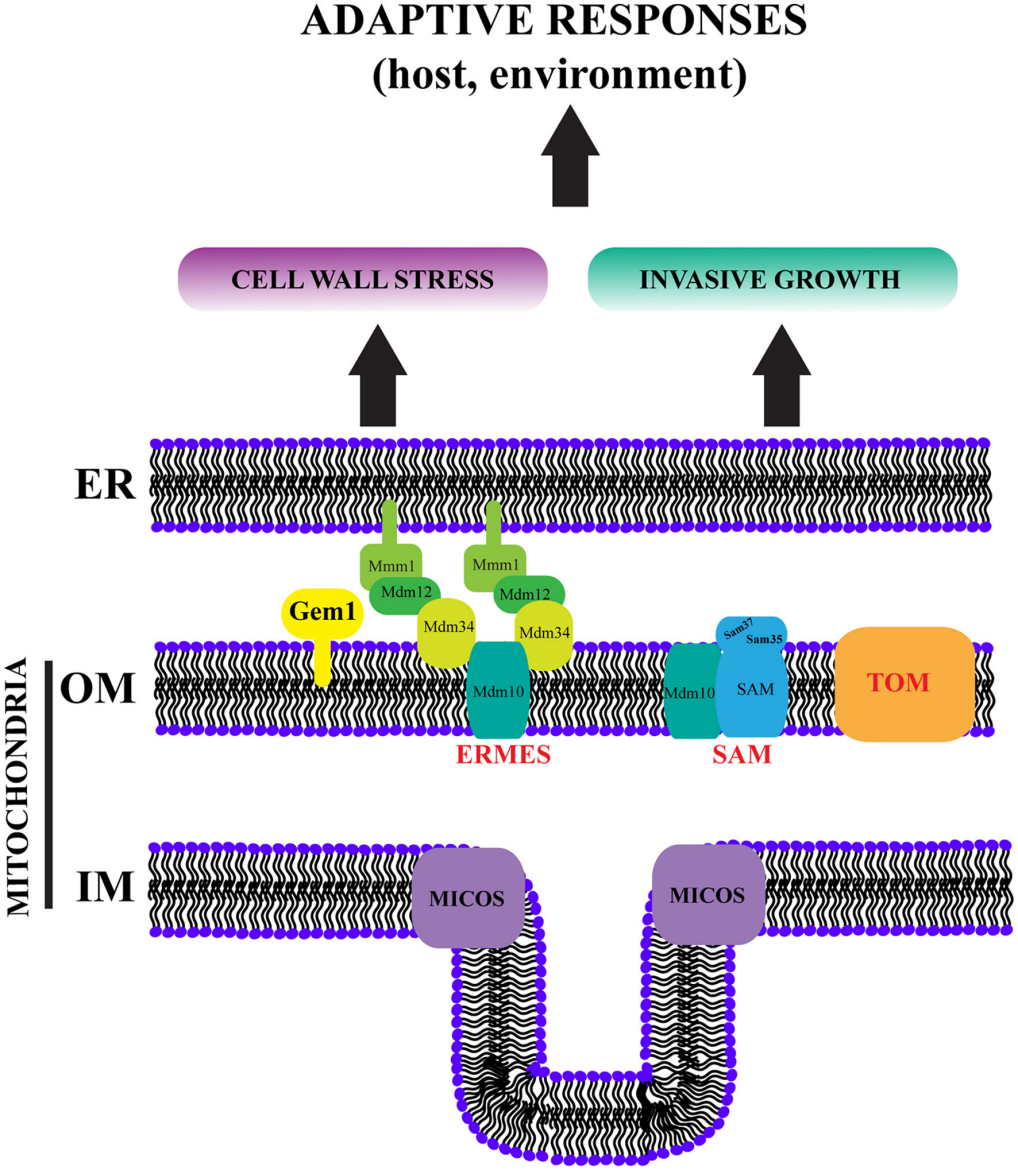
ERMIONE controls virulence behaviors in pathogenic fungi. In *S. cerevisiae* an ER-Mitochondria Organizing Network (ERMIONE) has been proposed to consist of the ERMES, SAM, TOM and MICOS complexes (shown here), and additional mitochondrial and ER proteins and complexes, as well as the vacuole-mitochondria tether vCLAMP, which are not depicted here for simplicity (van der Laan et al., 2012; Wideman and Muñoz-Gómez, 2016). ERMIONE is proposed to be a central hub for orchestrating mitochondrial biogenesis, lipid homeostasis, organelle dynamics and inter-organellar interactions. Based on our studies of Gem1 (this study), as well as the SAM and ERMES complexes in *C. albicans* (Qu et al., 2012; Tucey et al., 2016), and the study of ERMES in *A. fumigatus* (Geißel et al., 2017), we propose that ERMIONE plays a crucial role in the environmental adaptation of pathogenic fungi, which ultimately drives their virulence by controlling stress responses and invasive growth. Therefore, disruption of ERMIONE holds promise as antifungal therapy. ER: endoplasmic reticulum, OM: outer membrane, IM: inner membrane

In conclusion, our study defines a novel role for mitochondrial morphology factors in cell wall stress responses and invasive growth of *C. albicans*. In addition to Gem1 and ERMES (this study and Tucey et al., 2016), we have previously shown that the mitochondrial SAM complex is also needed for proper mitochondrial morphology and cell wall structure in *C. albicans* (Qu et al., 2012). Moreover, similarly to what we report here for ERMES and Gem1, it was the invasive growth ability of hyphae that was most strongly defective in the *sam37*Δ*/*Δ mutant (Qu et al., 2012). In *S. cerevisiae* SAM and ERMES complex share the Mdm10 subunit (Meisinger et al., 2004; Ellenrieder et al., 2016), and both are members of the ERMIONE organizing center for mitochondrial function (van der Laan et al., 2012). Moreover, the *S. cerevisiae* SAM complex interacts with the mitochondrial import complex TOM in ERMIONE (Wenz et al., 2015), and SAM subunits contact the ERMIONE-nucleating mitochondrial inner membrane complex MICOS (Harner et al., 2011). Overall the functions of SAM, ERMES and Gem1 in mitochondrial biogenesis and dynamics are conserved between *S. cerevisiae* and *C. albicans* (Hewitt et al., 2012; Qu et al., 2012; Tucey et al., 2016), and therefore it can be predicted that ERMIONE might exist in *C. albicans* as well. We propose that in addition to being a central organizing structure for mitochondrial activity, ERMIONE plays key roles in fungal virulence-related biology, particularly in regards to cell wall stress responses and invasive growth behaviors (Figure 7). These activities are crucial for environmental adaptation, and in the case of pathogenic fungi, for survival in host niches and upon antifungal treatments (Figure 7). Therefore, targeting ERMIONE-regulated pathway is highly promising as a future antifungal strategy.

## Author contributions

Conceptualized the project: BK, TMT, and AT. Performed experiments: BK, TMT, and TL. Assisted and provided advice on infection experiments: SN and PB. Analyzed the data: BK and TMT. Wrote the paper: BK and AT.

### Conflict of interest statement

The authors declare that the research was conducted in the absence of any commercial or financial relationships that could be construed as a potential conflict of interest.

## Supplementary material

Figure S1 Protein alignment of *C. albicans* Gem1, the human ortholog MIRO1 (hMIRO1) and human MIRO2 (performed with MUSCLE MUltiple Sequence Comparison by Log-Expectation v3.8.31).

Figure S2 Total cellular phospholipids were extracted from the indicated strains and separated by thin layer chromatography (TLC). Standards were used to identify cardiolipin (CL), phosphatidylserine (PS), phosphatidylethanolamine (PE), and phosphatidylcholine (PC).

Figure S3 Activation of the Cek1 signaling pathway in the *gem1*Δ*/*Δ mutant in response to cell wall stress. Cells were grown as described in Figure 3A, and phospho-Cek1 and actin detected as described in the section Materials and Methods. Five independent experiments (Exp1-Exp5) were performed. One is shown in Figure 3A and the other four are shown here. These experiments were used for quantification of the relative phospho-Cek1/Act1 levels at the 120 min post caspofugin treatment, and the quantification data is shown in Figure 3B. Exp6 was not used for quantification due to no detectable phospho-Cek1 band in the *gem1*Δ*/*Δ mutant. The control strain in this experiment was the completemented strain (+*GEM1*).

Table S1 Primers used in this study.
