# Supplementary figures and images for: The Mitochondrial GTPase Gem1 Contributes to the Cell Wall Stress Response and Invasive Growth of Candida albicans
Source: Front Microbiol. 2017 Dec 20;8:2555. doi: 10.3389/fmicb.2017.02555 (PMC5742345; doi:10.3389/fmicb.2017.02555)

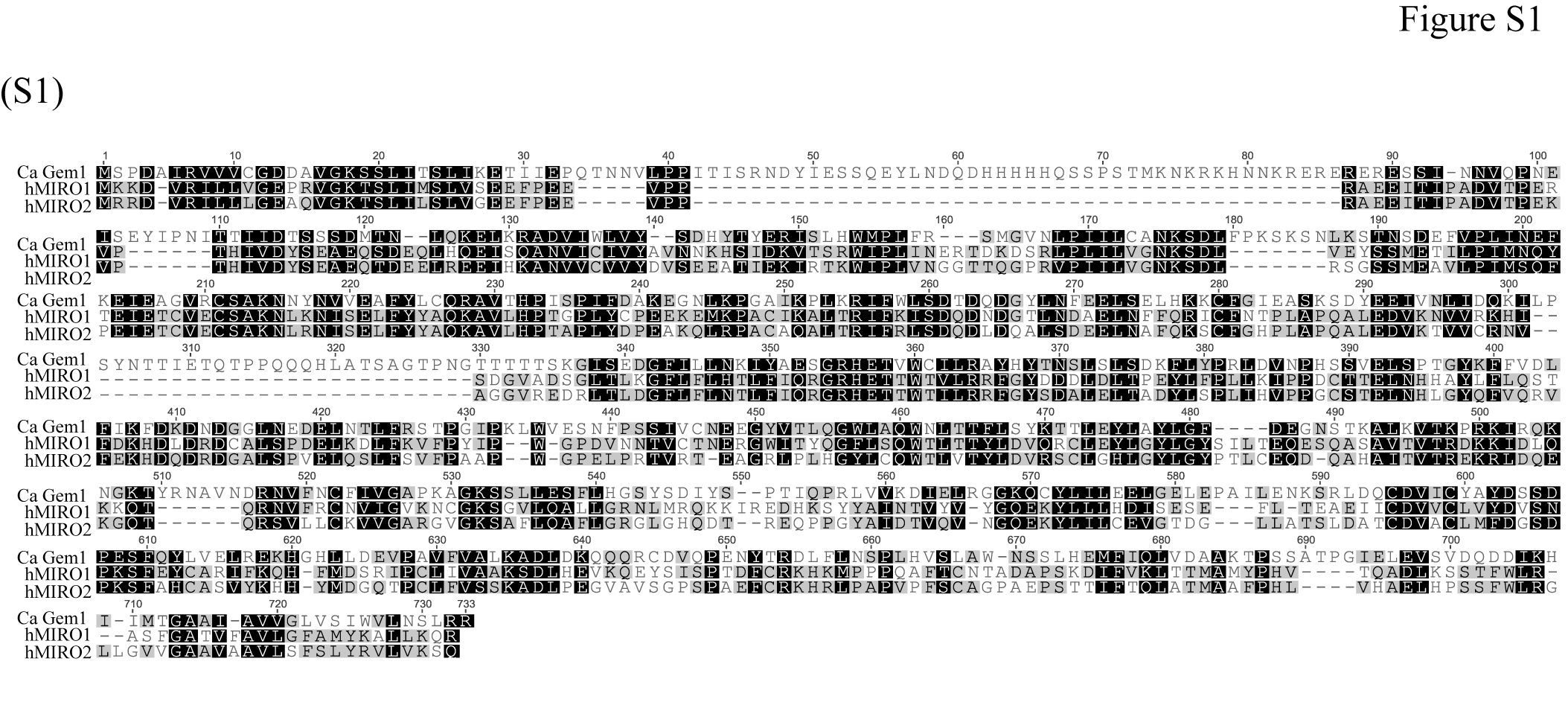

Supplement: Figure S1 — Protein alignment of C. albicans Gem1, the human ortholog MIRO1 (hMIRO1) and human MIRO2 (performed with MUSCLE MUltiple Sequence Comparison by Log-Expectation v3.8.31). [file Image1.TIF]

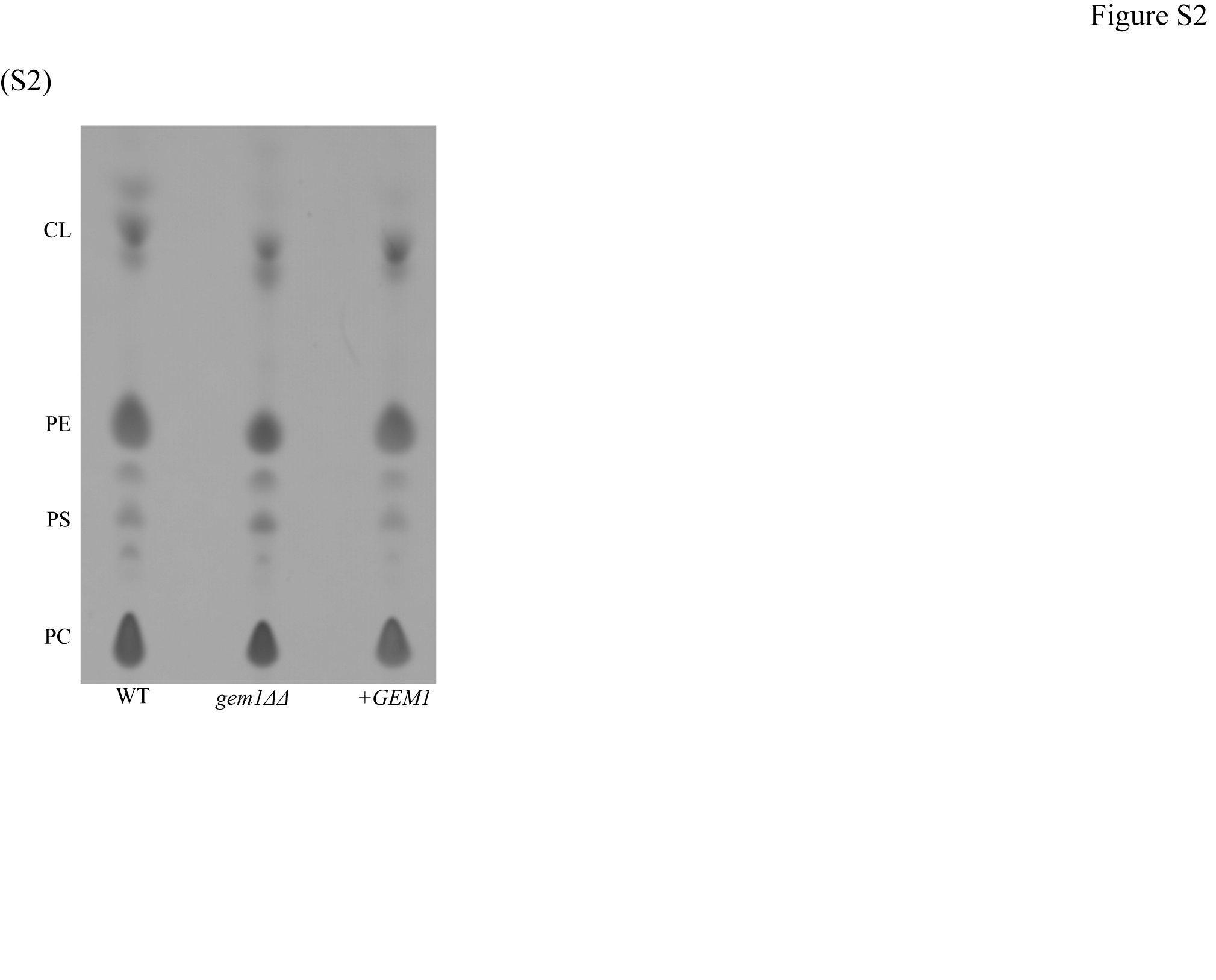

Supplement: Figure S2 — Total cellular phospholipids were extracted from the indicated strains and separated by thin layer chromatography (TLC). Standards were used to identify cardiolipin (CL), phosphatidylserine (PS), phosphatidylethanolamine (PE), and phosphatidylcholine (PC). [file Image2.TIF]

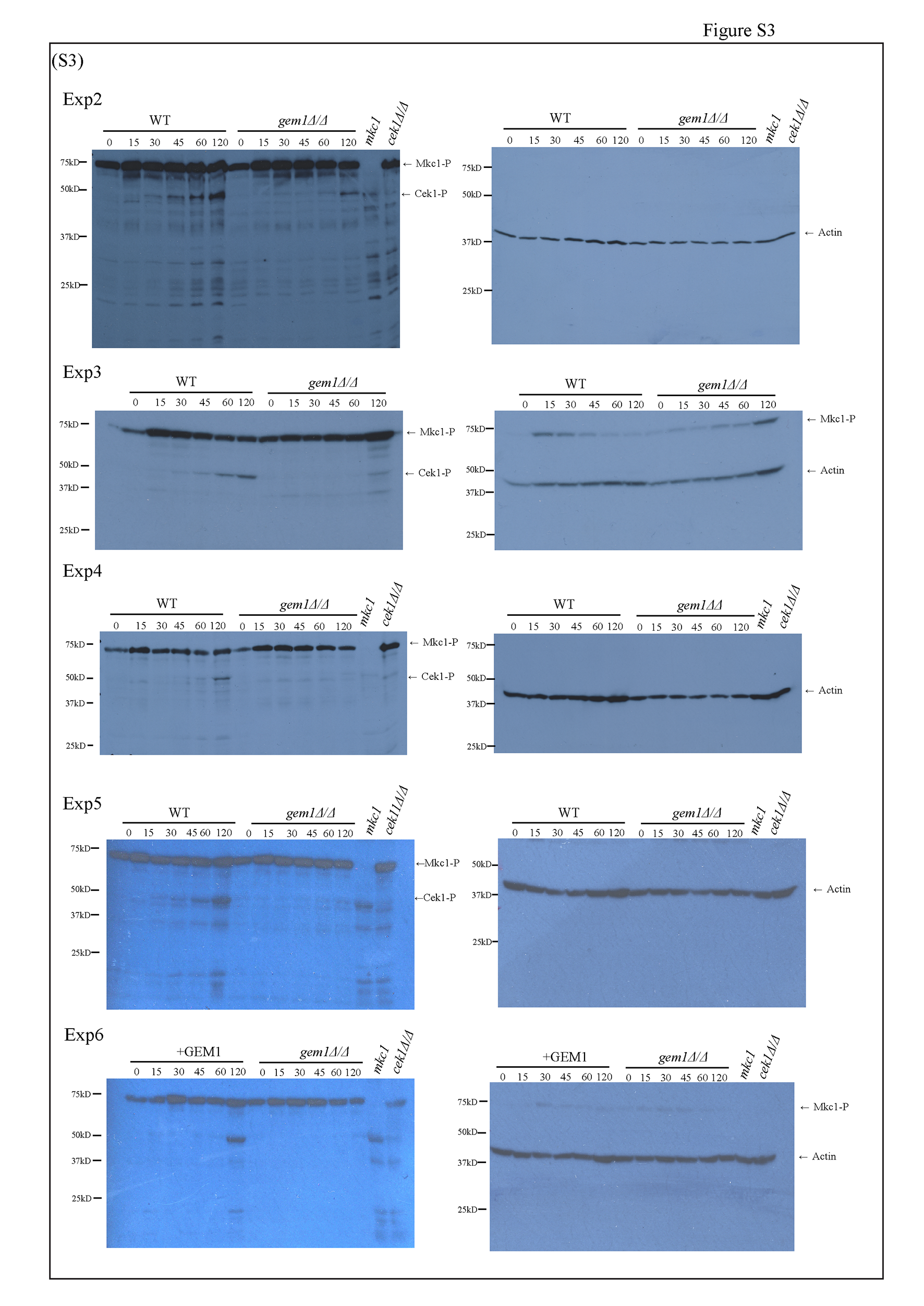

Supplement: Figure S3 — Activation of the Cek1 signaling pathway in the gem1Δ/Δ mutant in response to cell wall stress. Cells were grown as described in Figure 3A, and phospho-Cek1 and actin detected as described in the section Materials and Methods. Five independent experiments (Exp1-Exp5) were performed. One is shown in Figure 3A and the other four are shown here. These experiments were used for quantification of the relative phospho-Cek1/Act1 levels at the 120 min post caspofugin treatment, and the quantification data is shown in Figure 3B. Exp6 was not used for quantification due to no detectable phospho-Cek1 band in the gem1Δ/Δ mutant. The control strain in this experiment was the completemented strain (+GEM1). [file Image3.tif]
