# Supplementary material for: The Mitochondrial GTPase Gem1 Contributes to the Cell Wall Stress Response and Invasive Growth of Candida albicans
Source: Front Microbiol. 2017 Dec 20;8:2555. doi: 10.3389/fmicb.2017.02555 (PMC5742345; doi:10.3389/fmicb.2017.02555)
Supplement: Table S1 — Primers used in this study. [file Table1.PDF]

Table S1. Primers used in this study

GEM1 KO fw	agcaaaacatttttttttttggtgttgagaatgatcttacaattgaattatccataataaaatccagtgaaaaca aaccctcttgcacaacttcctaaaatttcccagtcacgacgttgtaaaac
GEM1 KO rev	tgatgagatggtgcgacaaatattttttttctttggctcttatacaaatccaaaaacagtaatttgtggacgccg cattcattcattctttcatttatttagtggaattgtgagcggata
GEM1 complementation fw	ttcacacaggaaacagctatgaccatgattacgccaagctagcactcaagtcgagaaagt
GEM1 complementation rev	tcgaccatatgggagagctcccaacgcgttggatgcatagattatagatgtatatatgta
CEK1 KO fw	agc cta ttt ttt ttt aaa ata att ttt ttt ttc aga tta att gaa tat ttc gac cac gtc atc aat aga aat cgc tac tac tac tta ctt aat aca aat gtt tcc cag tca cga cgt t
CEK1 KO rev	caa caa caa tta tgc taa atc tac aac aac tac caa gcc caa cct ata gtt ttt agt tta gtt tag ttt agt tta gtt tag ctt aac tta gct tga cct ctg tgg aat tgt gag cgg at
CEK1 HA fw	gat aaa atc cca gaa gat ttt ttc gat ttt gat aaa atg aaa gat caa tta aca att gaa gat ttg aaa aaa ttg tta tat gaa gag att atg aag cca tta ggt cga cgg atc ccc ggg tac
CEK1 HA rev	caa caa caa tta tgc taa atc tac aac aac tac caa gcc caa cct ata gtt ttt agt tta gtt tag ttt agt tta gtt tag ctt aac tta gct tga cct ctt cga tga att cga gct cgt t
MDM12 KO fw	cgacttattaaattttcataagtaaagtcattttgaagtgtataacgttctctctttaccaaccgatgacaacac ccagttaacgcaagcatcattataatttcccagtcacgacgttgtaaaac
MDM12 KO rev	gctattactatgtaataatttgatttctcaaccatattgacctgatatcgactttctttgtagcaattcatttatgtc catttagcacattgtggtgattagtggaattgtgagcggata
MDM12 MET rev	ggagtgagatatttttgaattgctgatccaaaaactcttttatcgattgattgatcgtgtcatcaattgttaattg attccaattaatatcaaatgacatgttttctggggagggtatttacttt
MDM12 complementation fw	ttcacacaggaaacagctatgaccatgattacgccaagctaatgtaatgaaatgcattgt
MDM12 complementation rev	tcgaccatatgggagagctcccaacgcgttggatgcatagactttgtctcaactgaatac
qCEK1 fw	caggctcaggctcaagctca
qCEK1 rev	ggctgctgcctgttgttgtt
q18S RNA fw	ggatttactgaagactaactactg

q18S RNA rev	gaacaacaaccgatccctagt
qSCR1 fw	tttagcataaccactggagggaag
qSCR1 rev	gagttgcaacactagataccgcact
qMMM1 fw	gactaccttcattatggcctagaact
qMMM1 rev	tagctgcactcgtaccgtttac
qMDM10 fw	accctgctgtgattcccaaa
qMDM10 rev	tgctgctgataatccaggac
